# Supplementary material for: Rising infrastructure inequalities accompany urbanization and economic development
Source: Nat Commun. 2025 Jan 30;16:1193. doi: 10.1038/s41467-025-56539-w (PMC11782700; doi:10.1038/s41467-025-56539-w)
Supplement: Supplementary file 1 — Supplementary Information [file 41467_2025_56539_MOESM1_ESM.pdf]

Supplementary Information

Rising infrastructure inequalities accompany urbanization and economic development

Bhartendu Pandey[1,2*], Christa Brelsford[3], Karen C. Seto[2]

[1] Geospatial Science and Human Security Division, National Security Sciences Directorate, Oak Ridge National Laboratory, TN, USA.

[2] Yale School of the Environment, Yale University, New Haven, CT, USA.

[3] Analytics, Intelligence, and Technology Division, Los Alamos National Laboratory, Los Alamos, NM, USA.

*Corresponding Author
Email: pandeyb1@ornl.gov

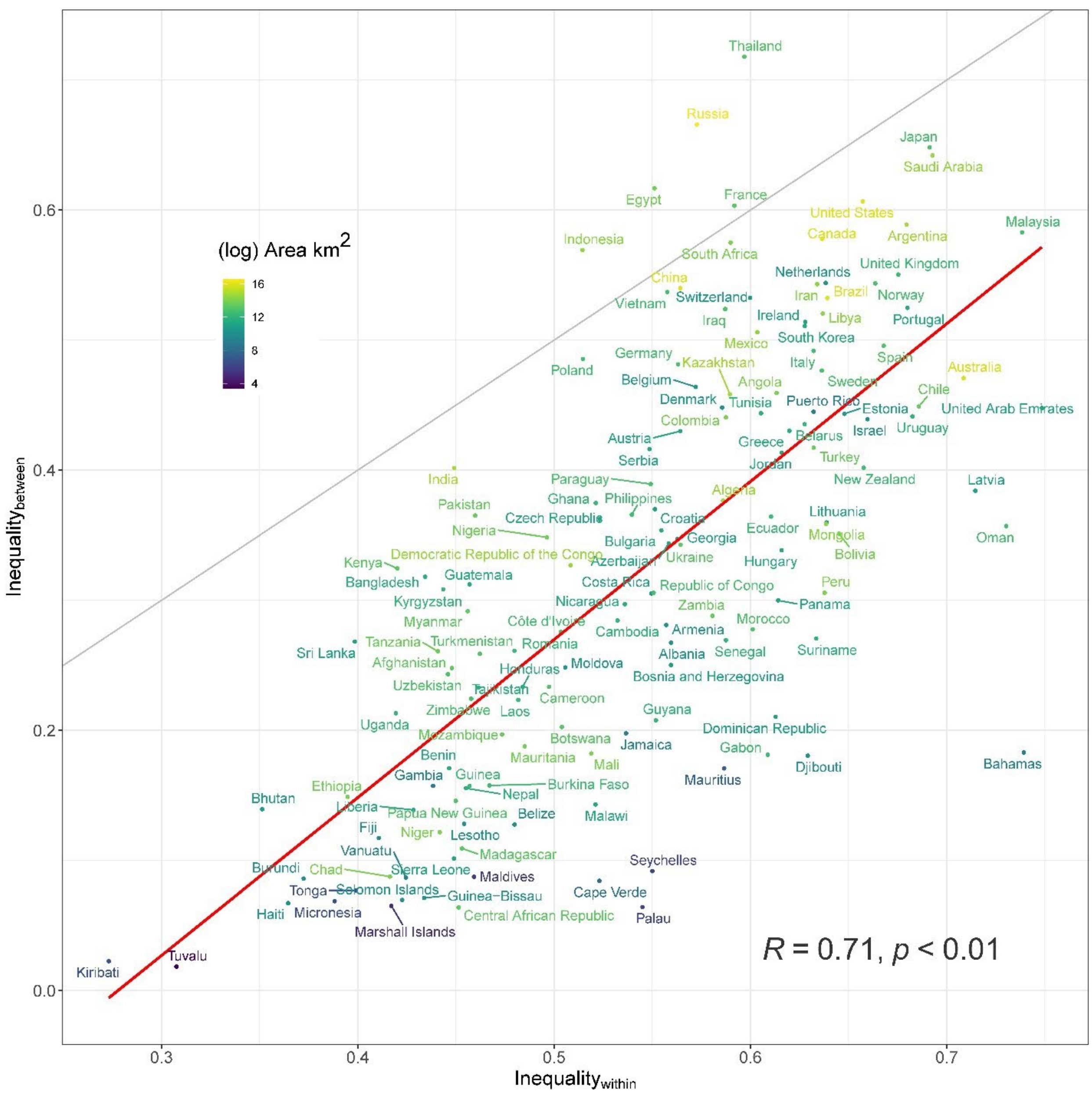

Fig. S1: Within-region (WR) and between-region (BR) inequality across 147 countries in the year 2015, estimated using a 0.5° lattice grid. Island nations and some countries with a significant land area (such as the United States, Russia, and Canada) have more significant deviations from the average relationship (red line).

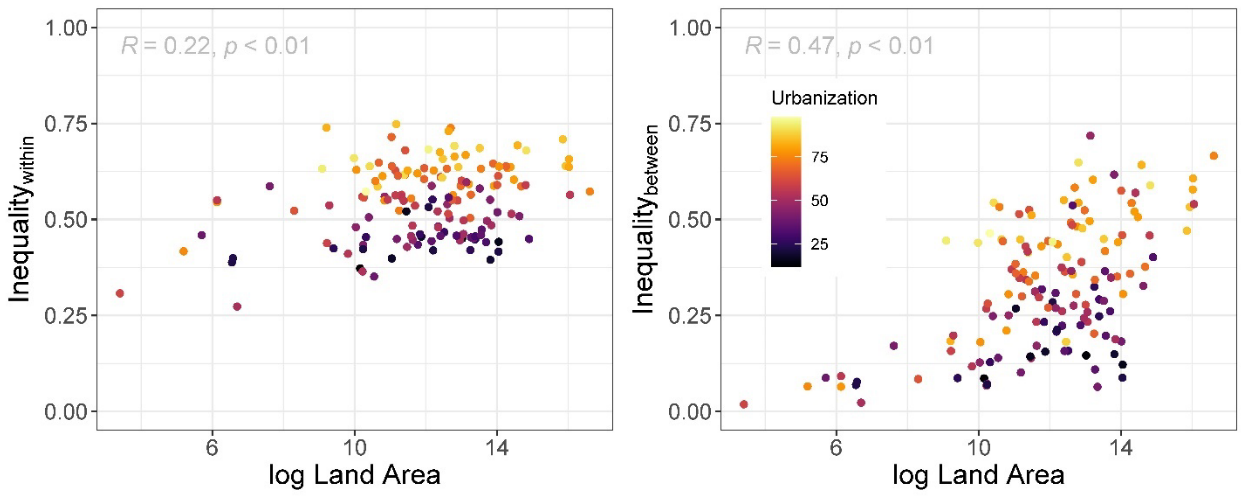

Fig. S2: Within-region (WR) (left) and between-region (BR) (right) inequality estimated for the year 2015 estimated using a 0.5° lattice grid and (log) land area (km^2) across 147 countries. Gray text shows Spearman's correlation coefficients (*R*).

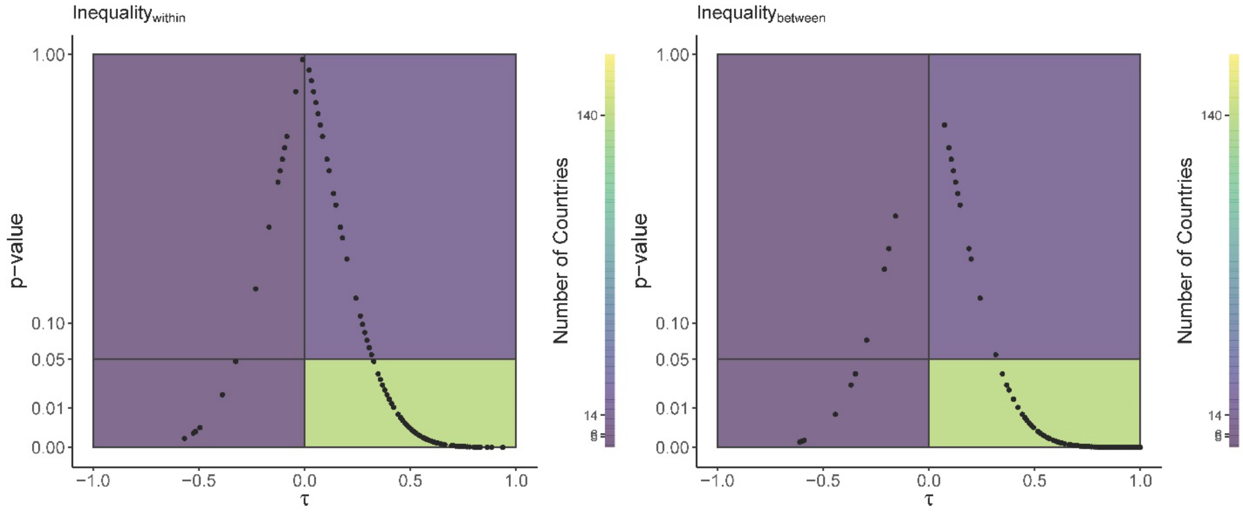

Fig. S3: Figure shows Kendall rank correlation coefficients (τ) for trends in WR ($\text{Inequality}_{\text{within}}$) and BR ($\text{Inequality}_{\text{within}}$) inequalities estimated using a 0.5° lattice grid and corresponding statistical-significance results from the Mann-Kendall trend test for 165 countries.

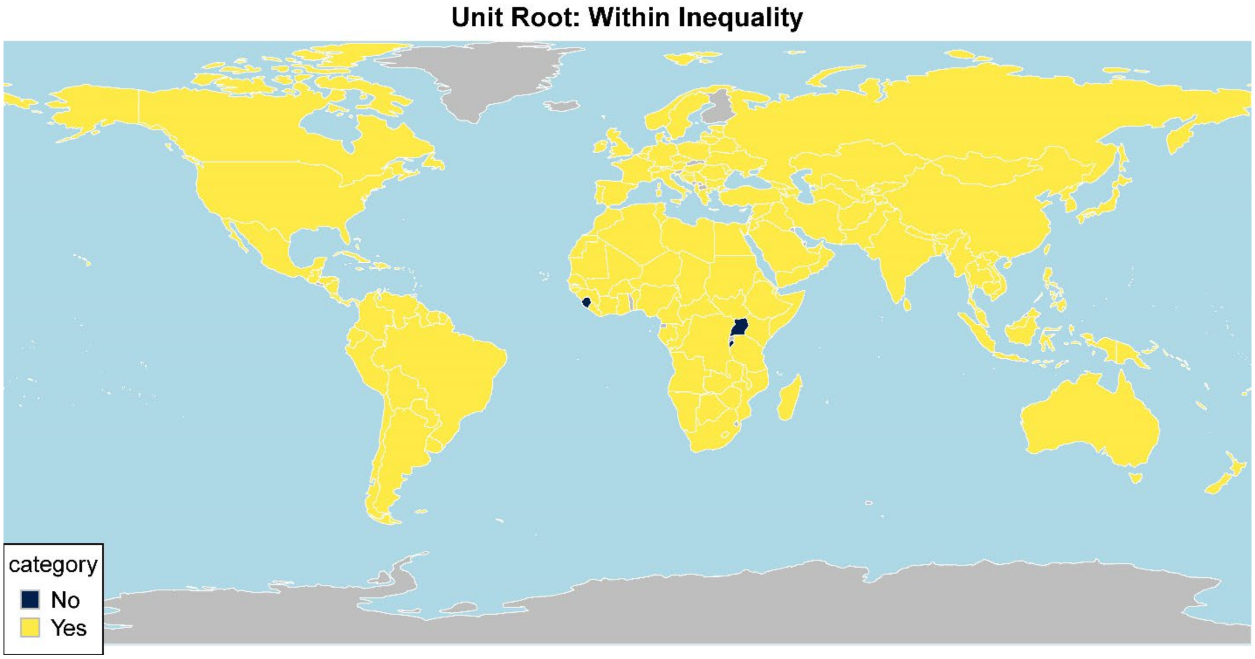

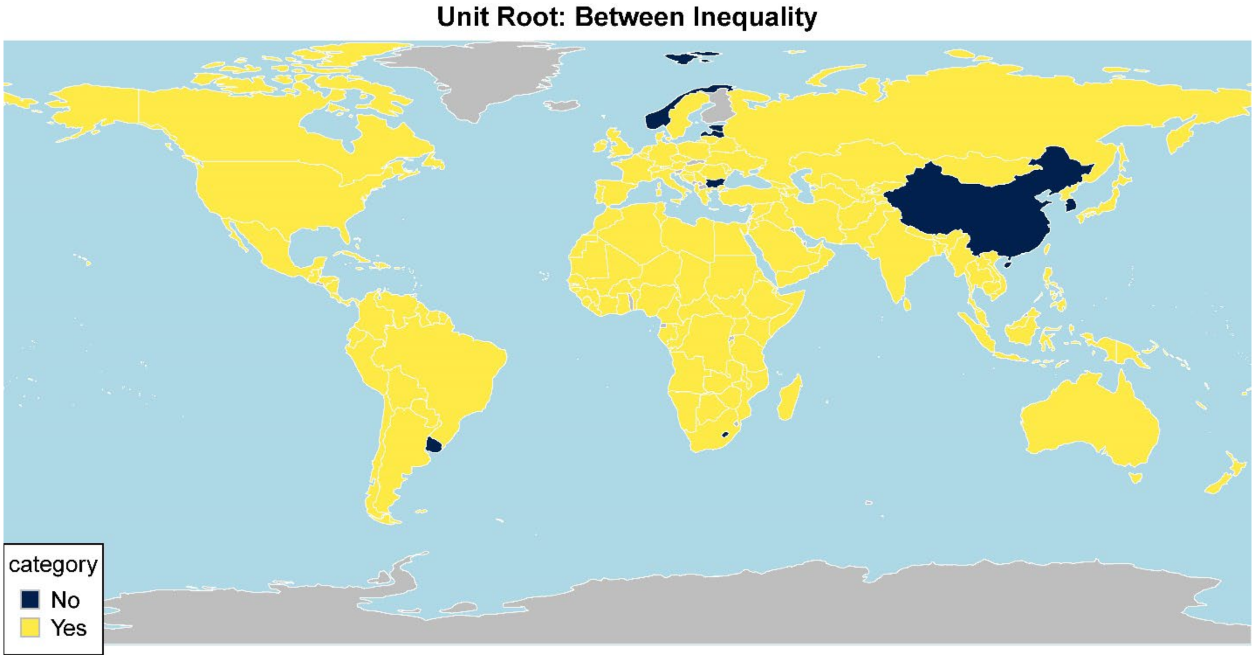

Fig. S4: Augmented Dickey-Fuller (ADF) test results for unit root's presence in within-region (top) and between–region inequality time-series, at the 0.05 significance level. The test indicates unit root in between-region (within-region) inequality time series, estimated using a 0.5° lattice grid, for 154 (162) countries out of 165 countries. Data for national boundaries depicted in this figure is from the open source global administrative areas (GADM) dataset version 3.6[1]. Boundary data for Austria is by the Government of Austria, licensed under CC BY-SA 2.0 (https://creativecommons.org/licenses/by-sa/2.0/deed.en), obtained from GADM (https://gadm.org). This figure is licensed under CC BY-SA 2.0.

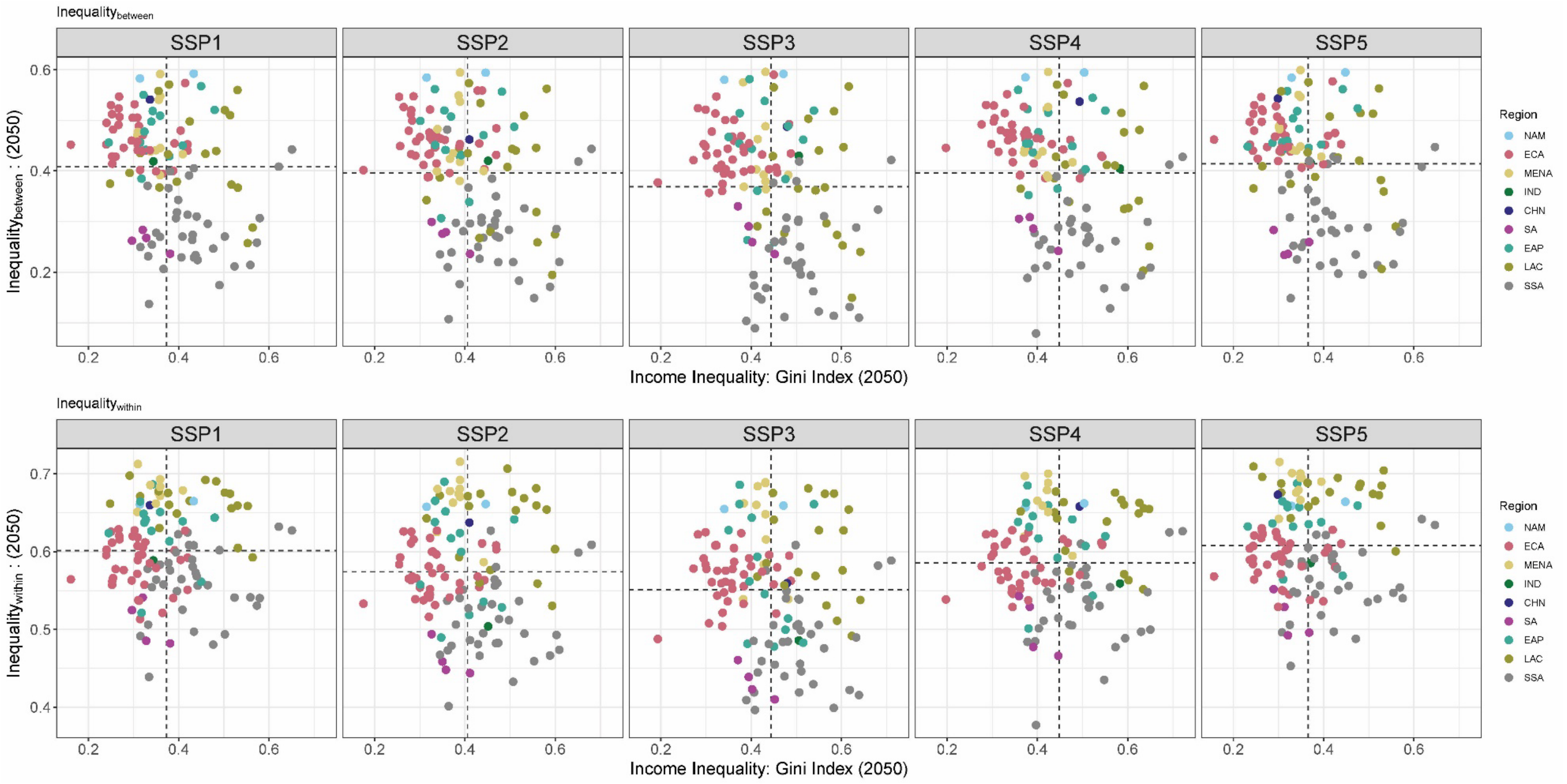

Fig. S5: Comparing forecasted 2050 between (Inequality$_{between}$) and within (Inequality$_{within}$) region infrastructure inequality levels with income inequality levels under the five shared socioeconomic pathways(SSP) for 121 countries classified into nine regions (a): sub-Saharan Africa (SSA), Latin America and the Caribbean (LAC), South Asia excluding India (SA), East Asia and Pacific excluding China (EAP), Middle East and North Africa (MENA), Europe and Central Asia (ECA), North America (NAM), India (IND), and China (CHN).

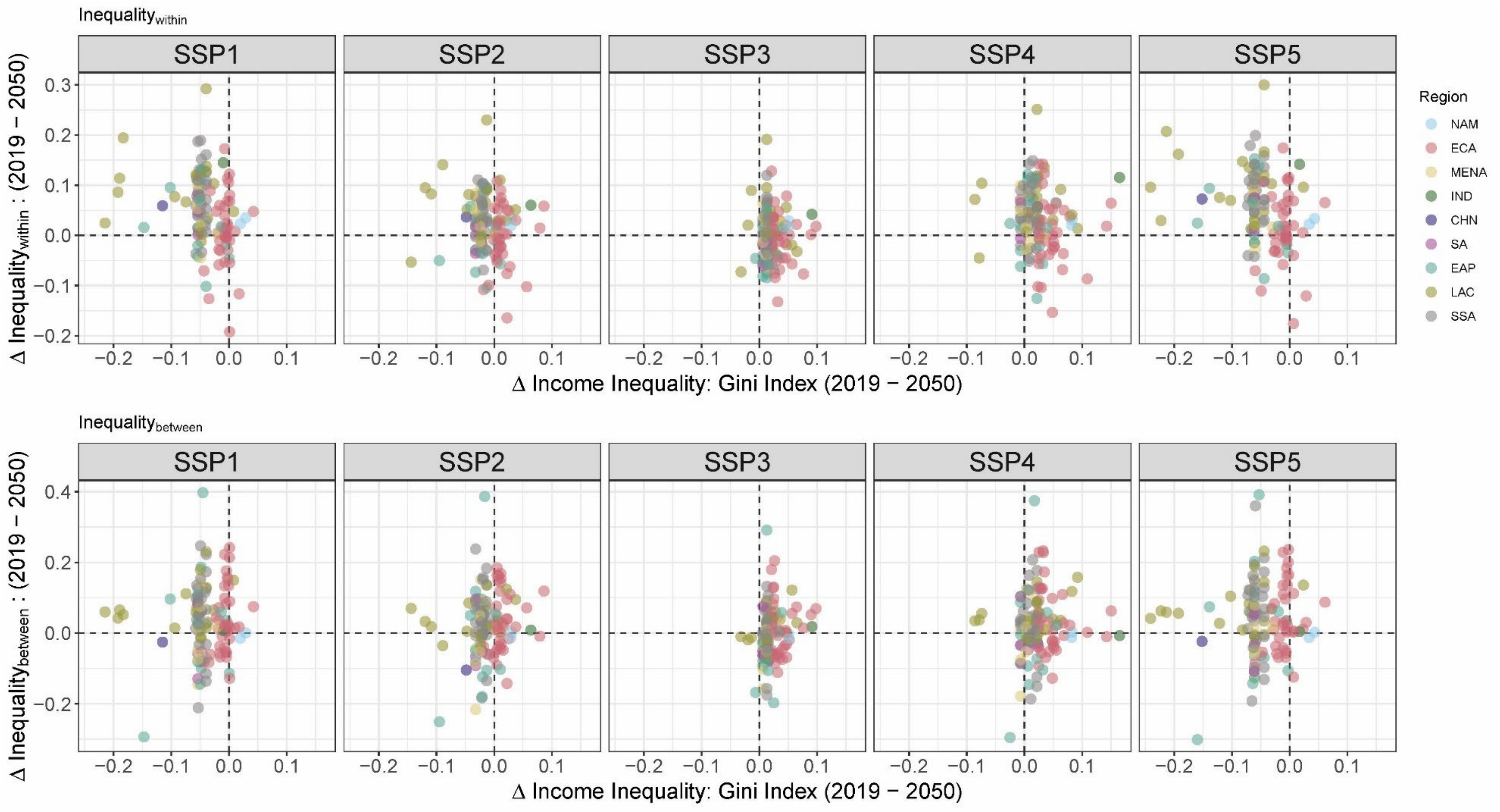

Fig. S6: Comparing forecasted between ($Inequality_{between}$) and within ($Inequality_{within}$) region infrastructure inequality changes with income inequality changes (2019-2050) under the five shared socioeconomic pathwasys (SSP) for 121 countries classified into nine regions (a): sub-Saharan Africa (SSA), Latin America and the Caribbean (LAC), South Asia excluding India (SA), East Asia and Pacific excluding China (EAP), Middle East and North Africa (MENA), Europe and Central Asia (ECA), North America (NAM), India (IND), and China (CHN).

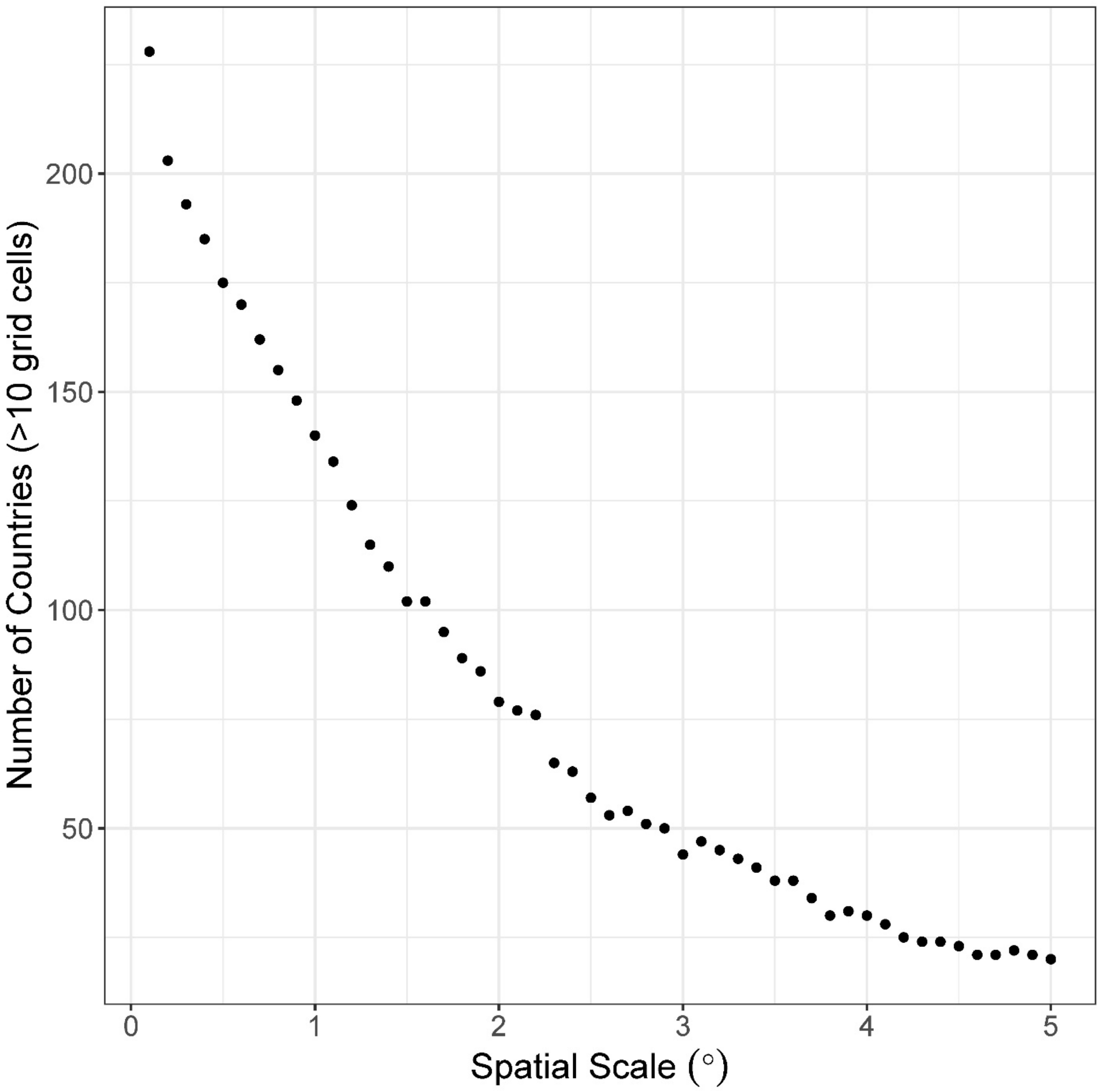

Figure S7: Number of countries with more than ten grid cells obtained at varying spatial scales.

Table S1: Ordinary least squares (OLS) regression for within-region and between-region inequalities across 147 countries in 2015, estimated using a 0.5° lattice grid.

	$Inequality_{within}$	$Inequality_{between}$
Urbanization-(log) GDP per capita (PC1)	0.057***	0.080***
	(0.003)	(0.005)
(log) Land Area (km^2)	0.011***	0.038***
	(0.002)	(0.003)
Constant	0.412***	-0.126***
	(0.030)	(0.034)
F Statistic (df = 2; 144)	170.897***	177.814***
Observations	147	147
R^2	0.706	0.712
Adjusted R^2	0.702	0.708
Residual Std. Error (df = 144)	0.053	0.089
Note:		***p<0.01 (Robust Standard Errors)

Table S2: Within-region inequality Analysis of Variance (ANOVA) results across 147 countries in 2015, estimated using a 0.5° lattice grid.

Within-Region Inequality

Variable	Sums of Squares	F value	p-value	Variation Explained (%)
Urbanization-(log) GDP per capita	0.86	313.531	<0.01	63.98
(log) Land Area (km^2)	0.09	32.519	<0.01	6.64
Residuals	0.40			

Table S3: Between-region inequality Analysis of Variance (ANOVA) results across 147 countries in 2015, estimated using a 0.5° lattice grid.

Between-Region Inequality

Variable	Sums of Squares	F value	p-value	Variation Explained (%)
Urbanization-(log) GDP per capita	1.83	229.18	<0.01	45.80
(log) Land Area (km^2)	1.02	127.25	<0.01	25.43
Residuals	1.15			

Table S4: Ordinary least squares (OLS) regression for changes in within- and between-region inequality.

	Δ $Inequality_{within}$		Δ $Inequality_{between}$	
	(1)	(2)	(3)	(4)
Δ Urbanization	0.002^{***}	0.002^{***}	0.003^{***}	0.002^{*}
	(0.001)	(0.001)	(0.001)	(0.001)
Δ (log) GDP per-capita	0.043^{***}	0.042^{***}	0.068^{***}	0.046^{***}
	(0.011)	(0.012)	(0.011)	(0.011)
Δ (log) Sum of Lights		0.005		0.064^{***}
		(0.013)		(0.016)
Constant	0.033^{***}	0.032^{***}	0.027^{***}	0.012^{*}
	(0.007)	(0.008)	(0.007)	(0.007)
F Statistic (df = 3; 140)	11.054***	7.4159***	20.636***	23.016***
Observations	144	144	144	144
R^2	0.165	0.166	0.259	0.412
Residual Std. Error	0.048 (df = 141)	0.048 (df = 140)	0.049 (df = 141)	0.044 (df = 140)
Note:				*p**p***p<0.01 (Robust Standard Errors)

Table S5: Error-correction model estimates for between-region inequality with urbanization (share of urban population to the total population) and sum of lights as covariates. Estimates correspond to a balanced panel of 143 countries and 19 time periods (2000-2019).

$Inequality_{between}$	Mean Group		Pooled Mean Group		Dynamic Fixed Effects	
	Estimate	*Std. Error*	*Estimate*	*Std. Error*	*Estimate*	*Std. Error*
Constant	-0.776***	0.153	-0.455***	0.038	-0.204***	0.015
**Long-run**						
Urbanization	0.006	0.009	0.003***	0.000	0.004***	0.001
Log Sum of Lights	0.093***	0.014	0.141***	0.004	0.134***	0.010
**Short-run**						
ECT	-0.563***	0.028	-0.269***	0.021	-0.127***	0.011
Δ Urbanization	0.046	0.056	0.001	0.031	-0.001	0.002
Δ Log Sum of Lights	0.004	0.004	0.023***	0.003	0.055***	0.001
***Significant at 0.01 level						
**Significant at 0.05 level						

Table S6: Error-correction model estimates for between-region inequality with economic development—log gross domestic product (GDP)—and sum of lights as covariates. Estimates correspond to a balanced panel of 143 countries and 19 time periods (2000-2019).

$Inequality_{between}$	Mean Group		Pooled Mean Group		Dynamic Fixed Effects	
	Estimate	*Std. Error*	*Estimate*	*Std. Error*	*Estimate*	*Std. Error*
Constant	-0.704***	0.068	-0.429***	0.041	-0.249***	0.017
**Long-run**						
Log GDP	0.270	0.261	0.037***	0.005	0.068***	0.012
Log Sum of Lights	0.097***	0.035	0.189***	0.007	0.127***	0.009
**Short-run**						
ECT	-0.402***	0.028	-0.168***	0.015	-0.130***	0.011
Δ Log GDP	-0.019**	0.009	0.003	0.008	-0.008	0.005
Δ Log Sum of Lights	0.012***	0.004	0.030***	0.004	0.055***	0.001
***Significant at 0.01 level						
**Significant at 0.05 level						

Table S7: Error-correction model estimates for within-region inequality with urbanization (share of urban population to the total population) and sum of lights as covariates. Estimates correspond to a balanced panel of 143 countries and 19 time periods (2000-2019).

$\text{Inequality}_{\text{within}}$	Mean Group		Pooled Mean Group		Dynamic Fixed Effects	
	Estimate	*Std. Error*	*Estimate*	*Std. Error*	*Estimate*	*Std. Error*
Constant	-0.148	0.206	-0.161***	0.014	-0.109***	0.025
**Long-run**						
Urbanization	0.022	0.018	0.001***	0.000	0.004***	0.001
Log Sum of Lights	-0.394	0.420	0.066***	0.004	0.046***	0.006
**Short-run**						
ECT	-0.682***	0.024	-0.488***	0.023	-0.409***	0.016
Δ Urbanization	-0.016	0.076	-0.004	0.031	-0.009***	0.003
Δ Log Sum of Lights	-0.050***	0.007	-0.050***	0.007	-0.007***	0.002
***Significant at 0.01 level						
**Significant at 0.05 level						

Table S8: Error-correction model estimates for within-region inequality with economic development—log gross domestic product (GDP)—and sum of lights as covariates. Estimates correspond to a balanced panel of 143 countries and 19 time periods (2000-2019).

$\text{Inequality}_{\text{within}}$	Mean Group		Pooled Mean Group		Dynamic Fixed Effects	
	Estimate	*Std. Error*	*Estimate*	*Std. Error*	*Estimate*	*Std. Error*
Constant	-0.497***	0.108	-0.216***	0.012	-0.227***	0.028
**Long-run**						
Log GDP	0.025	0.039	0.024***	0.004	0.058***	0.008
Log Sum of Lights	0.115**	0.052	0.069***	0.003	0.043***	0.006
**Short-run**						
ECT	-0.579***	0.024	-0.393***	0.022	-0.402***	0.016
Δ Log GDP	-0.056**	0.025	-0.032	0.020	-0.019	0.010
Δ Log Sum of Lights	-0.055***	0.008	-0.042***	0.007	-0.006**	0.003
***Significant at 0.01 level						
**Significant at 0.05 level						

Table S9: Magnitude of changes in between- and within-region inequality from 2019 to 2050 across world regions.

Region	SSP1	SSP2	SSP3	SSP4	SSP5
NAM	0.03	0.03	0.02	0.03	0.03
SA	0.03	0.02	0.05	0.03	0.04
ECA	0.04	0.04	0.02	0.04	0.05
MENA	0.04	0.03	0.02	0.03	0.05
CHN	0.06	0.11	0.09	0.06	0.08
SSA	0.09	0.05	0.01	0.05	0.10
LAC	0.12	0.08	0.04	0.09	0.13
IND	0.15	0.06	0.05	0.12	0.14
EAP	0.14	0.11	0.08	0.13	0.15
Note:	Change magnitude (C) is calculated using changes along between-region (ΔI_b) and within-region (ΔI_w) inequality: $C = \sqrt{(\Delta I_b)^2 + (\Delta I_w)^2}$.				

Supplementary References

1. GADM. Database of Global Administrative Areas (Version 3.6). (2018).
